# Case Report: Misdiagnosis of cellular cervical adenomyoma

**DOI:** 10.3389/fonc.2025.1584995

**Published:** 2025-10-03

**Authors:** Yuting Wang, Shuyuan Tian, Xiaoyu Han, Yingzi Xu

**Affiliations:** ^1^ Department of Ultrasound, Tongde Hospital Affiliated to Zhejiang Chinese Medical University, Hangzhou, Zhejiang, China; ^2^ Department of Pathology, Tongde Hospital Affiliated to Zhejiang Chinese Medical University, Hangzhou, Zhejiang, China

**Keywords:** cervical adenomyoma, cellular uterine leiomyoma, cervical cancer, case report, magnetic resonance imaging, ultrasound

## Abstract

**Background:**

Cellular cervical adenomyoma (CCA) is a rare benign tumor composed of proliferative smooth muscle cells and benign endocervical-type glands. Due to its nonspecific clinical and imaging features, CCA can mimic cervical malignancies, posing a diagnostic challenge.

**Case presentation:**

A 37-year-old woman presented with irregular vaginal bleeding. Pelvic examination revealed an approximately 3 cm friable mass on the posterior cervical lip. Transvaginal ultrasound (TVUS) showed a well-circumscribed, heterogeneous lesion with mild peripheral vascularity, favoring a cervical leiomyoma. In contrast, magnetic resonance imaging (MRI) demonstrated a 5 × 3 cm cervical mass with T2-weighted hyperintensity (T2WI), marked contrast enhancement, and diffusion restriction with a low apparent diffusion coefficient (ADC), raising concern for cervical carcinoma. The patient underwent hysteroscopic excision and diagnostic curettage. Histopathology confirmed a CCA, characterized by densely proliferative smooth muscle bundles with scattered benign endocervical-type glands, without cytologic atypia, mitotic activity, or stromal invasion. Ultrasound at 2 months showed no recurrence; at 12-month telephone follow-up, the patient reported regular menses without abnormal bleeding, although objective long-term surveillance remains necessary.

**Discussion:**

This case highlights the potential for CCA to mimic cervical cancer on MRI due to overlapping features. This case underscores the importance of considering CCA in the differential diagnosis of cervical masses with malignancy-like MRI features and highlights the need for histopathological confirmation to avoid misdiagnosis and overtreatment.

## Introduction

Cellular cervical adenomyoma (CCA) is an uncommon benign tumor of the cervix characterized by proliferative smooth muscle bundles admixed with benign endocervical-type glands (1). Population-level incidence estimates for CCA are unavailable; existing data derive from isolated case reports and small single-center series (2), with heterogeneous and often incomplete descriptions of imaging-pathology correlation. This underscores the need for clearer epidemiologic framing and standardized reporting of imaging features in CCA. Although the pathogenesis of CCA remains unclear, it is generally believed to be associated with prolonged effects of estrogen and progesterone (3). Histologically, CCA is characterized by a significant increase in smooth muscle cell density, while glandular and stromal components are relatively scarce (1, 4), making it prone to misdiagnosis as cervical cancer. Despite its clinical relevance, reports on CCA remain scarce-particularly those offering a comprehensive description of imaging misdiagnosis and preoperative diagnostic challenges. Recent studies, such as that by Ahvenainen et al. (5), have shown that cellular leiomyomas may exhibit metastatic potential and undergo malignant transformation, further supporting the need for heightened clinical vigilance regarding this tumor’s biological behavior.

Early differentiation of CCA from cervical carcinoma can be guided by imaging patterns: transvaginal ultrasound typically shows a well-circumscribed, heterogeneous cervical mass with mild peripheral vascularity and a clear interface with the cervical canal-features favoring a leiomyoma-like lesion-whereas MRI frequently demonstrates T2-weighted hyperintensity (T2WI), avid contrast enhancement, and diffusion restriction with a low apparent diffusion coefficient (ADC), a constellation that may mimic malignancy (6, 7). Key radiologic distinctions between CCA and cervical cancer-with common pitfalls—are summarized in **Table 1** to aid early differential diagnosis (8, 9). To bridge these gaps, the present work documents explicit discordance between ultrasound and MRI and correlates the malignancy-like MRI appearance with histopathological substrates (high smooth-muscle cellularity, glandular dispersion, and focal stromal/cystic change), providing practical cues to avoid overtreatment when imaging findings are incongruent.

**Table 1:** Imaging features of cellular cervical adenoma (CCA) and cervical cancer.

Imaging	CCA (benign)	Cervical cancer (malignant)	Common pitfalls/notes
US (margins and echotexture)	Well-circumscribed, heterogeneous; mild peripheral vascularity; clear interface with canal	irregular/infiltrative; heterogeneous, richly vascular	CCA cellularity can look solid; degeneration may mimic necrosis on US
MRI (T2 signal)	hyperintense or mixed (glandular/stromal components)	Typically iso- to hyperintense vs stroma	T2 hyperintensity is non-specific; both entities may look similar
MRI (contrast enhancement)	May enhance avidly (cellularity/vascularity)	Early strong enhancement common	Avid enhancement alone does not confirm malignancy
DWI/ADC	Diffusion restriction can occur; ADC may be low	Diffusion restriction typical; low ADC	Low ADC is not specific—correlate with histology

CCA, cellular cervical adenomyoma; US, Ultrasound; MRI, magnetic resonance imaging; DWI, Diffusion-Weighted Imaging; ADC, apparent diffusion coefficient.

Given these challenges, early diagnosis and differential diagnosis are crucial for the treatment and prognosis of patients, and long-term close follow-up is recommended (5). In this report, we present a case of CCA initially misdiagnosed as cervical cancer, with a focus on the diagnostic discrepancy between ultrasound and Magnetic resonance imaging (MRI) findings. By integrating clinical, radiological, histopathological, and immunohistochemical data, our study addresses key diagnostic pitfalls and expands the current understanding of this uncommon but clinically significant entity.

## Case presentation

**Figure 1:**
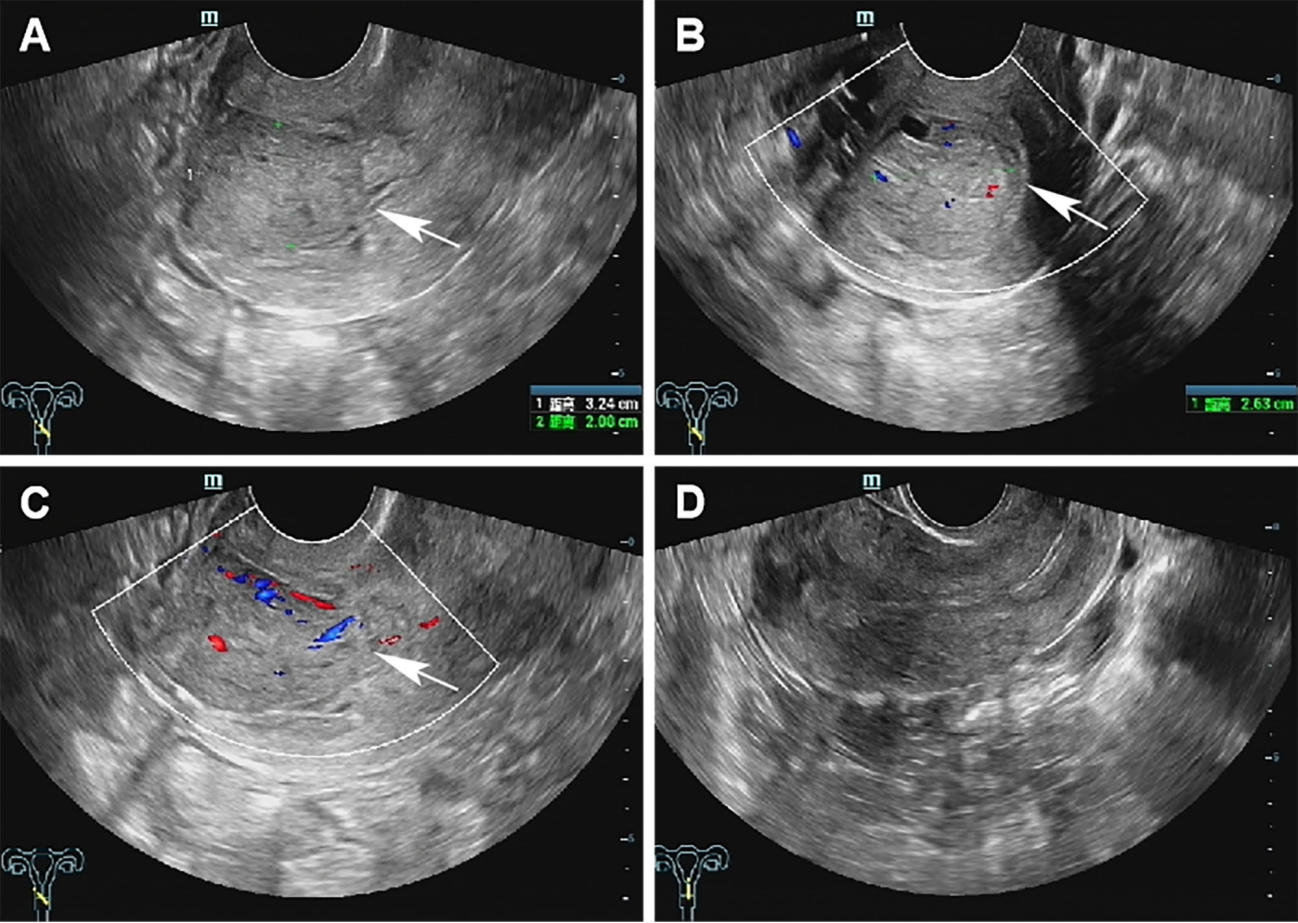
Preoperative and postoperative ultrasound images. **(A)** Transvaginal ultrasound longitudinal view revealed a medium-to-high echoic mass on the posterior lip of the cervix (white arrow). **(B)** Transvaginal ultrasound shows a transverse section image of the cervical mass (white arrow). **(C)** Color doppler flow imaging (CDFI) showed the cervical mass with slightly abundant blood flow signals (white arrow). **(D)** Transvaginal ultrasound revealed a normal uterus following adenomyomectomy.

**Figure 2:**
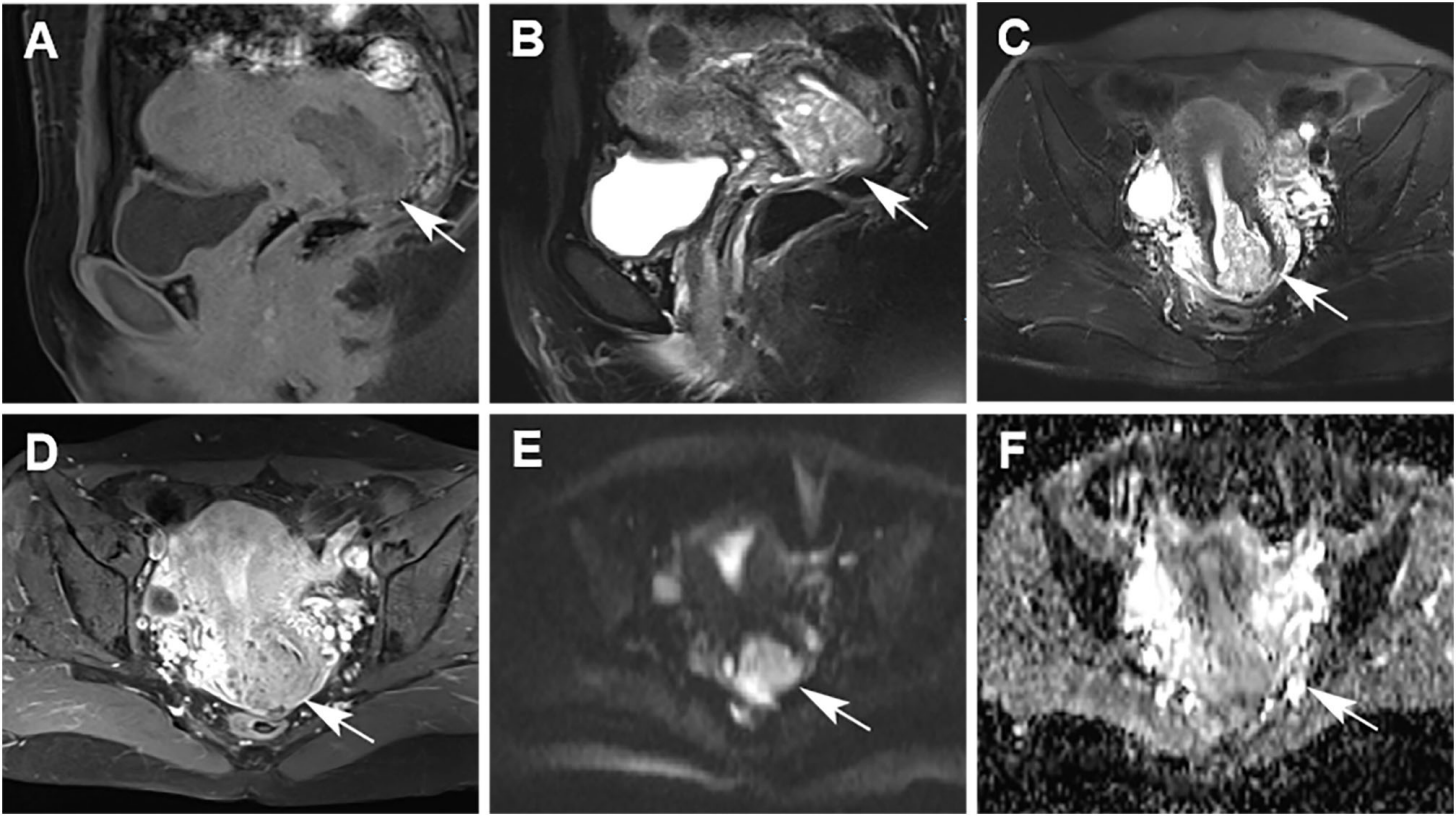
Magnetic resonance imaging (MRI) revealed a mass-like soft tissue tumor in the cervix. **(A)** MRI showed a slightly low signal on T1-weighted imaging (white arrow). **(B)** MRI showed a high signal on T2-weighted sagittal imaging (white arrow). **(C)** MRI showed a high signal on T2-weighted axial imaging (white arrow). **(D)** Contrast-enhanced magnetic resonance imaging (CE-MRI) showed that the lesion was significantly enhanced on contrast-enhanced scanning of T1-weighted imaging (white arrow). **(E)** High signal on diffusion-weighted imaging (DWI) (white arrow). **(F)** Reduced signal on apparent diffusion coefficient (ADC) (white arrow).

**Figure 3:**
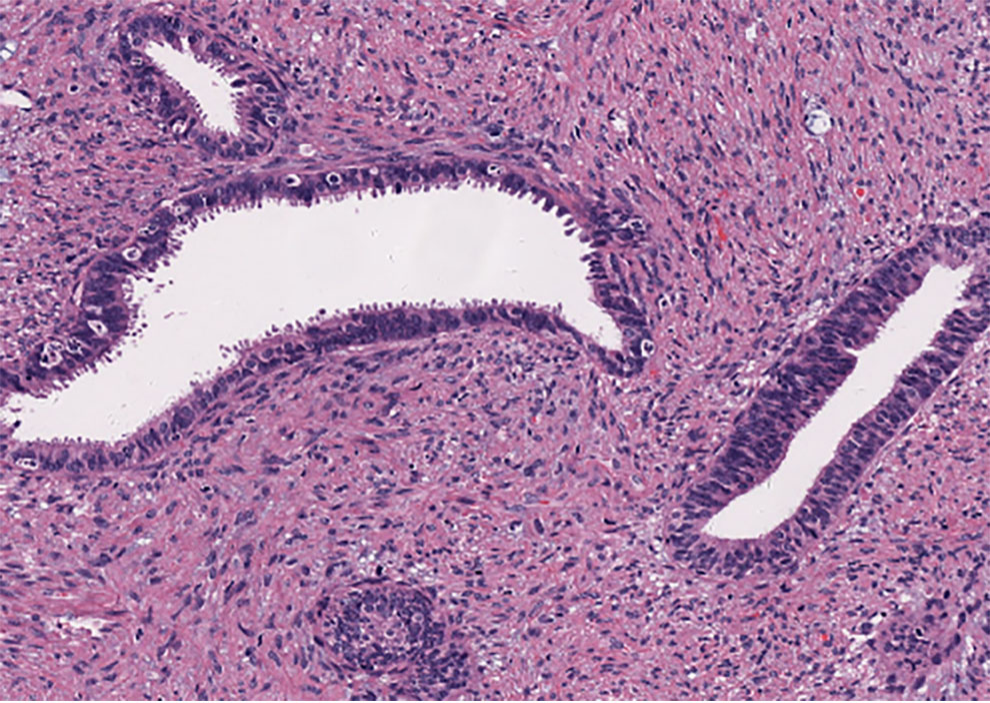
Histopathological of case: mass stained with hematoxylin and eosin (original magnification x 10).

**Figure 4:**
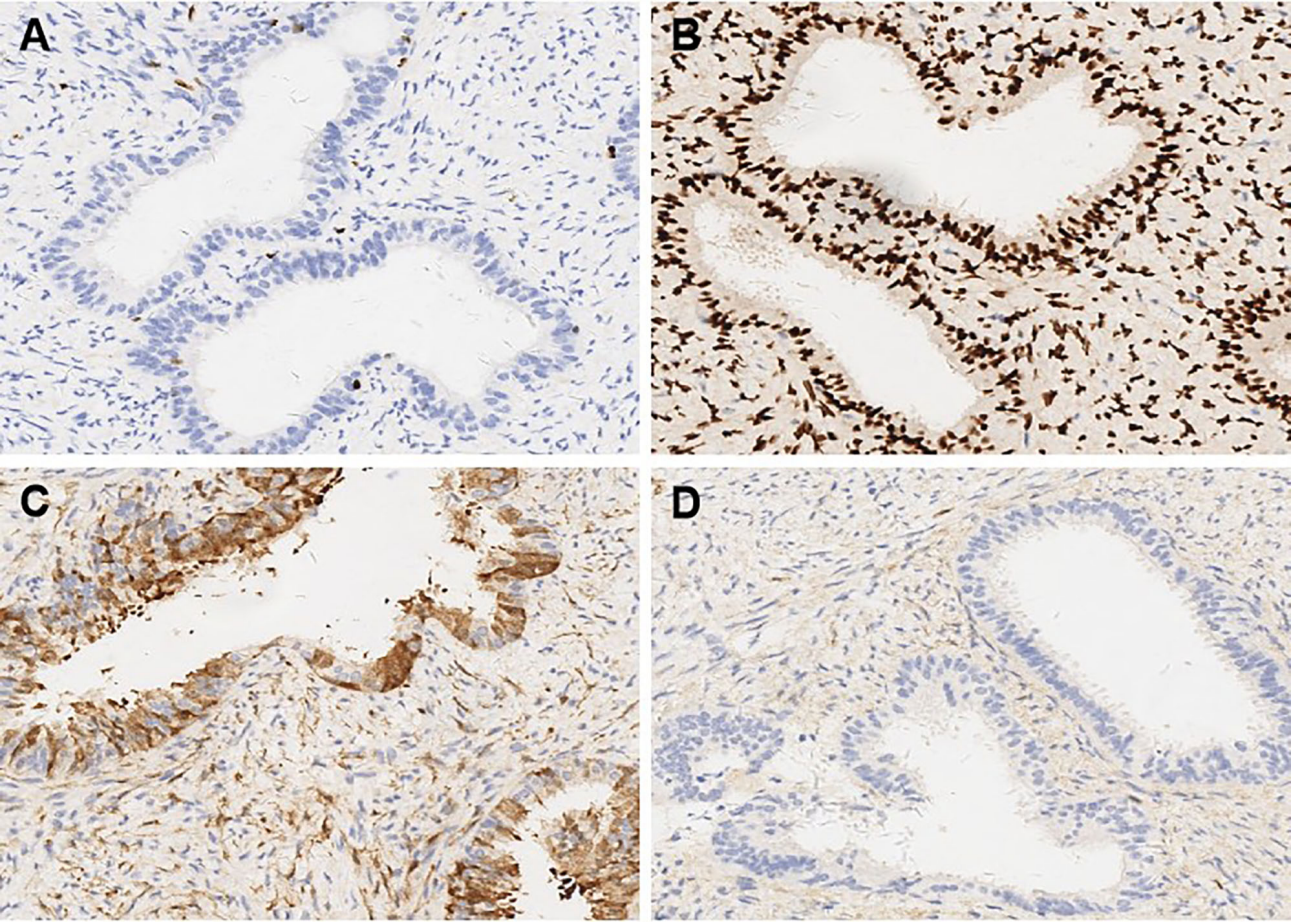
Immunohistochemical findings in cellular cervical adenomyoma (original magnification x 10). **(A)** The proliferation index of Ki-67 was 2%. **(B)** Estrogen Receptor (ER) showed positive expression in immunohistochemical staining. **(C)** P16 showed positive expression in immunohistochemical staining. **(D)** CD10 showed positive expression in immunohistochemical staining.

A 37-year-old married woman (gravida 2, para 1) presented to the gynecology department on June 7, 2023, with irregular vaginal bleeding lasting 23 days. Her last menstrual period was April 20, 2023. She had experienced regular menstruation since menarche at age 13, with a 25-day cycle and 5-day duration, reporting heavy flow without dysmenorrhea or pelvic discomfort. She had a surgical history of ectopic pregnancy in 2013 and no known family history of hereditary disease. Gynecological examination revealed a small amount of dark red blood in the vagina, and a cauliflower-like mass measuring 3×3 cm on the lower lip of the cervix, which was fragile in texture and associated with persistent bleeding. Ultrasound (US) showed a 3×2 cm mass with mixed echogenicity on the posterior lip of the cervix (**Figure 1A**). The mass had clear borders, a regular shape, and heterogeneous internal echogenicity, with a clear demarcation from the cervical canal (**Figure 1B**). Mild increased blood flow signals were observed around the mass (**Figure 1C**). The ultrasound results suggested cervical leiomyoma. However, based on the patient’s history and physical examination, the clinician suspected a malignant tumor. The patient underwent pelvic MRI, which revealed a soft tissue mass in the cervix measuring approximately 5×3 cm. The T1-weighted image (T1WI) showed slightly low signal intensity (**Figure 2A**), while the T2WI displayed high signal intensity (**Figures 2B, C**) and fat-suppressed T2 signals. Diffusion-weighted imaging (DWI) showed high signal intensity (**Figure 2E**), with a decreased ADC signal (**Figure 2F**). Contrast-enhanced scanning showed significant enhancement of the lesion (**Figure 2D**), leading to a diagnosis of cervical cancer. The ThinPrep cytology test (TCT) showed moderate cervical inflammation. The human papillomavirus (HPV) test result was negative. Serum tumor markers, including carbohydrate antigen 125 (CA125), cancer antigen 199 (CA199), and squamous cell carcinoma antigen, were all within normal ranges. Colposcopy-guided biopsy revealed chronic cervical inflammation, focal squamous epithelial hyperplasia of the cervical mucosa, and a mixed epithelial-mesenchymal tumor on the lower lip of the cervix. Immunohistochemistry indicated adenomyoma. The patient subsequently underwent hysteroscopic cervical lesion resection and diagnostic curettage. During hysteroscopy, severe cervical erosion was noted, with the cervical canal markedly enlarged and 70% of the mass located within the cervical canal. The mass was fragile in texture, measuring approximately 4×5 cm. The surgery proceeded smoothly, and the postoperative specimen confirmed the presence of a fragile cervical tumor. The final histopathological results showed cervical adenomyoma with active cellular proliferation (**Figure 3**). Immunohistochemical staining revealed positive results for smooth muscle actin (SMA), estrogen receptor (ER), P16, and CD10, with a Ki-67 proliferation index of 2% (**Figures 4A–D**). The patient was discharged 5 days postoperatively and recovered well. Two months after follow-up, no signs of recurrence or metastasis were observed (**Figure 1D**). During the follow-up period, the patient did not experience symptoms such as vaginal bleeding or abdominal pain, and estrogen and progesterone levels remained stable. At 12-month telephone follow-up, the patient reported regular menstruation with a 28-day cycle and 5-day duration, normal menstrual volume and color, and no symptoms of dysmenorrhea, abnormal vaginal bleeding, or pelvic discomfort.

## Discussion

CCA is an exceedingly rare benign tumor. Nonetheless, it may exhibit biologically aggressive behaviors, including recurrence and metastasis (1). Due to the nonspecific nature of its clinical presentation and imaging features, CCA is often misdiagnosed as cervical cancer. In this report, we present a case initially suspected as cervical cancer based on imaging findings. The patient underwent hysteroscopic excision of the cervical lesion, and subsequent histopathology confirmed a diagnosis of CCA. The short-term prognosis was favorable, with no evidence of recurrence or metastasis during the two-month follow-up.

Both US and MRI play critical roles in the preoperative assessment of cervical lesions. However, there are limited reports detailing the imaging features of CCA. In a recent case by Tien et al. (10), serial transvaginal US was used to monitor a recurrent cervical adenomyoma, which showed progressive enlargement with preserved benign histologic characteristics. In our case, US revealed a well-defined, heterogeneous mass with mild peripheral vascularity, suggestive of cervical leiomyoma. In contrast, MRI demonstrated high T2 signal intensity, strong contrast enhancement, and reduced ADC values—features more consistent with malignancy. This discordance contributed to the initial misdiagnosis of cervical cancer. Transvaginal ultrasound (TVUS) can assess tumor size and suspected stromal/parametrial involvement; pooled estimates report sensitivity/specificity around 62%/91% for parametrial invasion and 84%/80% for stromal invasion, with substantial heterogeneity across operators and techniques (11). MRI provides higher overall accuracy for local staging and stromal invasion and is emphasized in FIGO 2018 and ESGO/ESTRO/ESP updates; adding DWI/ADC improves delineation of highly cellular tumors (6, 12). PET-CT/PET-MRI add greatest value for nodal and distant staging rather than for differentiating benign from malignant cervical masses (7).

Several factors may have contributed to this diagnostic discrepancy. 1) The patient’s symptom of irregular vaginal bleeding and the friable cervical mass on examination prompted a clinical suspicion of malignancy, potentially biasing radiologic interpretation; 2) Both cervical cancer and CCA may present as high signal intensity on T2WI (13) and exhibit diffusion restriction on DWI with reduced ADC values (14); 3) The dense smooth muscle cellularity of CCA can mimic the solid appearance of malignant lesions; 4) Areas of degeneration within large CCAs may resemble necrosis seen in malignant tumors. These overlapping imaging features can largely be explained by the histopathological characteristics of CCA. The abundant proliferation of smooth muscle cells, along with scattered benign endocervical-type glands, results in a solid appearance on ultrasound and high signal intensity on T2WI. Localized cystic changes or stromal edema may further mimic tumor necrosis. Moreover, high cellular density and vascularity can lead to strong enhancement and diffusion restriction, which are typically interpreted as markers of malignancy. Recent literature has also emphasized the importance of correlating imaging findings with histopathological features in gynecological lesions that demonstrate MRI patterns suspicious for cancer. For instance, Salman et al. (15) reported that certain benign uterine masses with high stromal cellularity could show marked enhancement and low ADC values, contributing to misinterpretation. Taken together, these findings underscore the necessity of integrating imaging with histological confirmation to avoid diagnostic errors and prevent overtreatment. Intraoperative frozen section (IFS) can help prevent overtreatment in gynecologic oncology when an intact mass is available, but its performance depends on specimen type and sampling. Contemporary series report overall high specificity with variable sensitivity, which declines for small or fragmented samples and borderline lesions. In our case, hysteroscopic resection produced friable fragments, so we did not obtain IFS to avoid sampling error; instead, we relied on permanent sections for definitive diagnosis (16). In our case, hysterectomy was initially considered due to the lesion’s size and imaging features suggestive of malignancy. However, colposcopic biopsy subsequently indicated cervical adenomyoma, reducing the suspicion for cancer. The patient, aged 37 and desiring fertility preservation, strongly declined hysterectomy despite counseling on potential risks. After careful multidisciplinary evaluation and shared decision-making, hysteroscopic resection was chosen to preserve the uterus. Although complete resection was considered challenging due to the lesion’s size, the patient accepted the surgical plan after informed consent, and the procedure proceeded without complications.

From a histopathological perspective, CCA may present with overlapping imaging and clinical features similar to those of malignant cervical tumors, particularly adenocarcinoma and adenosarcoma. CCA typically appears as a well-circumscribed lesion composed of densely proliferative smooth muscle cells interspersed with scattered endocervical-type glands (10, 17, 18). These glands are usually lined by a single layer of bland columnar epithelium, without cytological atypia, mitotic activity, or stromal invasion (5). In contrast, cervical adenocarcinoma is characterized by architectural complexity, nuclear pleomorphism, increased mitotic activity, and invasive stromal infiltration (8). Although rare, cervical adenosarcoma demonstrates a biphasic pattern with benign epithelial elements and malignant stromal components, often accompanied by periglandular cuffing, increased stromal cellularity, and frequent mitoses (10). Immunohistochemistry plays a crucial role in differentiating CCA from malignancy. In CCA, the Ki-67 proliferation index is typically low—often less than 10%—in contrast to malignant cervical tumors where the index frequently exceeds 20% and can surpass 60% (3). In our case, the Ki-67 index was 2%, supporting a benign diagnosis. Additionally, ER staining has proven to be a valuable adjunct, as CCA glands are usually diffusely ER-positive, whereas malignant cervical tumors often lack ER expression (19). The strong ER positivity observed in our case further corroborates the diagnosis of CCA. Tien et al. (10) reported a case of recurrent cervical adenomyoma following supracervical hysterectomy, in which transvaginal ultrasound monitoring demonstrated progressive enlargement of the lesion while maintaining benign histologic characteristics. Similar cases were also reported by Yamamoto et al. (8), where imaging features mimicked cervical malignancy, but histopathological examination confirmed a diagnosis of benign cervical adenomyoma. Taken together, both previous literature and our current case emphasize the necessity of incorporating detailed histopathological and immunohistochemical evaluation into the diagnostic workflow to avoid overtreatment resulting from misinterpretation of imaging studies.

Currently, there is no standardized treatment protocol for CCA. The primary treatment for CCA is surgical excision (10, 17). The choice between local excision and total hysterectomy should be individualized, considering factors such as patient age, fertility desire, general health, and intraoperative pathology. Despite its benign nature, CCA may recur or metastasize in rare cases. For instance, Guraslan et al. (20) documented pelvic recurrence 10 years after total hysterectomy for cellular leiomyoma, and Ahvenainen et al. (5) reported a case with lung metastasis and malignant transformation. Therefore, long-term postoperative surveillance is crucial, regardless of the extent of surgery. In our case, the patient underwent hysteroscopic excision, with no recurrence at 2-month imaging follow-up. At 12-month telephone follow-up, she remained asymptomatic with regular menstruation and no signs of abnormal bleeding or pelvic discomfort. Nonetheless, objective imaging-based monitoring remains necessary to fully assess long-term outcomes.

The present case not only complements the limited existing descriptions of imaging features in CCA, but also contributes novel insights into its ultrasound presentation and highlights diagnostic challenges arising from discordant imaging interpretations. By presenting a complete diagnostic timeline—from initial clinical evaluation to imaging and histopathological confirmation—this report offers a more comprehensive framework for understanding and recognizing this rare entity. Nevertheless, a limitation of this case is the lack of advanced ultrasound techniques, such as contrast-enhanced ultrasound (CEUS) and shear-wave elastography (SWE), which could provide further value in the differential diagnosis of cervical lesions. In this case, MRI was performed immediately after transvaginal ultrasound on the same day due to strong clinical suspicion of malignancy. The subsequent MRI findings suggested cervical cancer, prompting the patient’s rapid admission and precluding additional ultrasound-based assessments. The absence of CEUS or SWE may have limited the comprehensiveness of imaging evaluation. Second, cases should consider incorporating such advanced techniques when feasible, to further enhance diagnostic specificity. Third, the hysteroscopic resection yielded multiple friable tissue fragments. Consequently, no macroscopic images could be obtained during surgery or at the time of pathological processing. Finally, long-term imaging surveillance was not performed at our institution. Although the patient remained asymptomatic at 12-month telephone follow-up and reported routine outpatient visits at a local hospital, objective imaging data beyond 2 months were unavailable, limiting our ability to fully assess recurrence risk.

## Conclusion

In conclusion, CCA is an extremely rare benign tumor that may closely mimic cervical cancer on imaging, leading to potential misdiagnosis and overtreatment. The varying sensitivity and specificity of different imaging modalities underscore the importance of correlating radiological findings with histopathological and immunohistochemical confirmation, which remains the gold standard for diagnosis. Given its potential for recurrence and malignant transformation, long-term follow-up is essential. Clinicians and radiologists should maintain a high index of suspicion when evaluating cervical masses and be familiar with the imaging features of CCA to avoid unnecessary interventions. Multidisciplinary collaboration is critical to achieving accurate diagnosis and optimal patient outcomes.

## Data Availability

The original contributions presented in the study are included in the article/supplementary material. Further inquiries can be directed to the corresponding author.
